# Effect of a Positive Photo Appreciation Program on Depressive Mood in Older Adults: A Pilot Randomized Controlled Trial

**DOI:** 10.3390/ijerph15071472

**Published:** 2018-07-12

**Authors:** Masumi Ishihara, Tami Saito, Takashi Sakurai, Hiroyuki Shimada, Hidenori Arai

**Affiliations:** Center for Gerontology and Social Science, National Center for Geriatrics and Gerontology, 7-430 Morioka-cho, Aichi 474-8511, Japan; t-saito@ncgg.go.jp (T.S.); tsakurai@ncgg.go.jp (T.S.); shimada@ncgg.go.jp (H.S.); harai@ncgg.go.jp (H.A.)

**Keywords:** positive emotion, positive intervention, photography, positive psychotherapy, depression, positivity, randomized controlled trial, mood improvement, combination of multiple dimensions program, older adults

## Abstract

Safer and more effective programs are required to cope with an increasing number of older people with depression. Hence, we developed the Positive Photo Appreciation (PPA) program. A three-month pilot randomized controlled trial was conducted with healthy Japanese individuals aged 65–84 years, assigned to a PPA group (*n* = 28) or Photo Correspondence Education (PCE) (control group) (*n* = 27). We used the Center for Epidemiologic Studies Depression Scale (CES-D) score as the primary outcome measure. Secondary outcome measures, among others, were cognitive function and positive emotion. Data collected at baseline and post-intervention were analyzed using a linear mixed-effect model. Over 80% of the participants in the PPA group completed and were satisfied with the program. Compared with the PCE group, the CES-D score in the PPA group significantly improved (main effect of group: *t* = −4.30, *p* < 0.001; interaction effect of group by time: *t* = 4.39, *p* < 0.001), with an effect size of *d* = 1.23. Additionally, a positive significant interaction effect of group by time was found in the Positive and Negative Affect Schedule (*t* = −2.33, *p* = 0.024). The PPA program might be promising for mitigating depressive mood in older adults.

## 1. Introduction

Aging populations are a global issue. The number of people aged 60 years or more is estimated to be 962 million, comprising 13% of the global population [1]. Furthermore, this number is expected to rise rapidly; it has been estimated that people aged 60 years or more will comprise more than 25% of the total population in most regions of the world by 2050 [1]. For instance, in Japan, which has the highest proportion of aging population in the world, 30% of the population is expected to be aged 65 years or more by 2025 [2].

Depression is a major disease burden in older adults [3]. Depression in older adults may be caused by negative mood, which is often triggered by negative life events such as bereavement, health decline, and anxiety about life [4]. In addition, recent studies have revealed that depression is also related to reduced vitality [5], and is a risk factor for dementia in older adults [6,7]. Because of the aging population, the importance of preventing and treating depression in older adults is, therefore, apparent, and the development of safe and effective non-pharmacological mood improvement programs is imperative.

Recent studies have shown the effectiveness of positive psychotherapy (PPT), an intervention based on positive psychology, on depressive symptoms [8,9,10,11,12,13,14]. PPT contrasts with standard interventions targeting depressive symptoms because it focuses on building positive emotions, character strength, engagement, and meaning to alleviate depression rather than directly targeting depressive symptoms [8,9]. A recent study showed that PPT enhances six core well-being processes: positive emotion, use of strength, optimism, self-compassion, resilience, and positive relations, and that these processes are possible mechanisms underlying improvements of mental well-being, anxiety, and depressive symptoms [15]. Additionally, other studies have reported that PPT significantly decreased the levels of mild-to-moderate depression and showed higher remission rates in patients with major depressive disorder than Treatment-As-Usual at the counseling and psychological services [8,9]. However, few PPTs have been developed for older adults despite their high affinity for positivity [16].

Therefore, we developed the Positive Photo Appreciation (PPA) program for older adults based on a combination of the multiple components of PPT mentioned above [8]. Studies have shown that the PPT or other types of positive psychology programs could provoke more significant improvements when they combined multiple components, such as positive emotion, engagement, and meaning, rather than using a single component [8,17,18,19,20,21]. We chose photography as a tool for the program because it is one of the popular learning activities or hobbies among older adults [22,23,24]. We considered photography (i.e., capturing photos, viewing pictures, conversations related to photos, etc.) as a positive and enjoyable activity and predicted that it would enhance three elements: positive emotion, engagement, and meaning, which would lead to the improvement of depression. This improvement would be elicited by the photography, collage production, appreciation, and discussion. In addition, we added “Three Good Things” and “Gratitude Visit” to our PPT program, based on the study of Seligman et al. [17] on the “five happiness exercises”, as they have been shown to effectively alleviate depression when used as part of PPT [8,17,25].

The purpose of this pilot study was to evaluate the feasibility of the PPA program and its effect on depressive mood in healthy older adults. As secondary outcomes, we evaluated changes in positive emotions, cognitive functions, and vitality. We hypothesized that cognitive function and vitality could be improved by alleviating depressive mood. Since this is the first study that examines the effect of this PPA program, we also calculated the effect size in preparation for future full-scale randomized controlled trials (RCT).

## 2. Materials and Methods

### 2.1. Study Design

**Figure 1:**
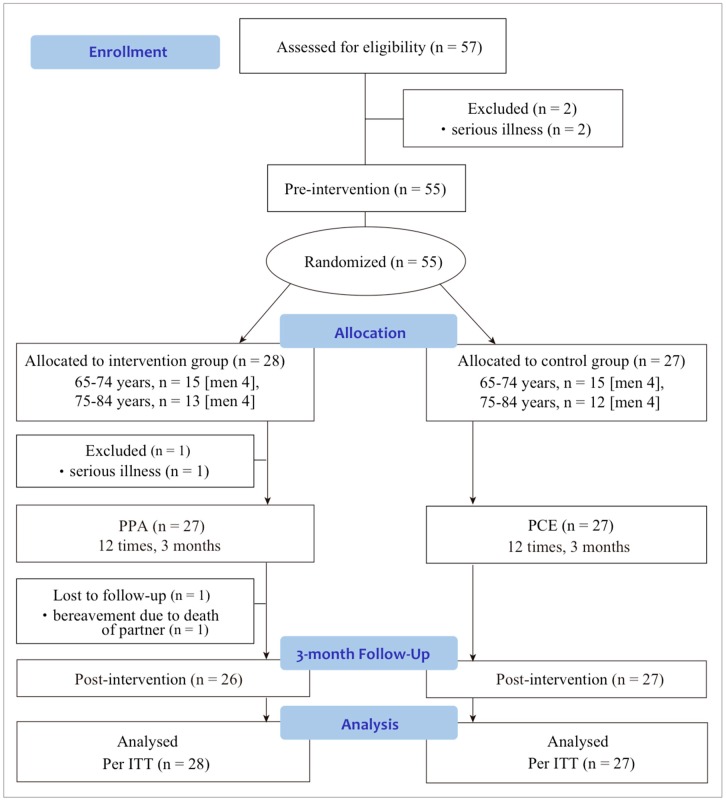
Flowchart of the pilot randomized controlled trial. Notes: PPA: Positive Photo Appreciation; PCE: Photo Correspondence Education; ITT: Intention-To-Treat.

This study used a standardized protocol of a three-month intervention in multiple facilities in the community. The study was a two-arm, non-blind (only analysis was conducted blind), parallel RCT. It was approved by the ethics committee of the National Center for Geriatrics and Gerontology in Japan (No. 935-2), and the protocol was in accordance with the Declaration of Helsinki. Assessments were performed before the start of the PPA program (baseline) and after intervention (follow-up). The study procedure flow diagram is shown in Figure 1.

### 2.2. Participants

Fifty-seven Japanese healthy, community-dwelling older adults (38 women, aged from 65 to 84 years) living in two municipalities were recruited between October 2016 and November 2016. We distributed recruitment fliers to our institution’s neighboring areas, or enlisted the help of social workers to recruit older adults. The following individuals were included: (1) healthy men and women aged 65–84 years, (2) those who could perform instrumental activities of daily living by themselves, and (3) those who provided written informed consent. The following individuals were excluded: (1) those who were receiving psychotropic drugs or anti-depressants (not including anti-anxiety and sleep-inducing drugs) at the time of recruitment; (2) those who had had cerebrovascular disease, myocardial infarction, or angina attack within the past 6 months; (3) those who had progressive diseases, such as cancer or advanced visual impairment; and (4) those who were judged unsuitable for the intervention by the principal investigator (e.g., those with serious illnesses, such as consciousness disorders). Information regarding medication and diseases was based on participants’ self-reports.

### 2.3. Study Settings

Since it was difficult for all the subjects to participate in the intervention at the same time, they were divided into 5 groups of 4–7 participants, and each group participated in the program, which was held at their nearest community center. The implementation period, content of implementation, implementer, and measurement time were the same across the groups.

### 2.4. Randomization

Participants were randomly allocated by sex and age category (65–74/75 years and above). Generation of the allocation sequence was performed using computer-generated random numbers, and the allocation ratio was 1:1. For blinding, each participant’s name was replaced by a number assigned by the intervention practitioner and a person unrelated to the study project.

### 2.5. Interventions

#### 2.5.1. PPA Program (Intervention Group)

The program was performed once a week for each group. Each session lasted 1.5 h (photography time: 30 min, appreciation time: 1 h). A total of twelve sessions, between December 2016 and March 2017, were completed. Collages were produced once every 4 sessions (production time: 1 h, appreciation time: 30 min).

In this study, we used a commercially available, compact digital camera as the intervention material. For the photography, participants captured photos of their choice after being taught how to operate the camera. They captured several pictures with the help of the facilitator, both inside and outside the facility.

Participants were instructed to capture pictures according to their preferences. In addition, they also took pictures of other participants smiling. For the collage production, participants created a collage using their own portrait and photos of the participants smiling, photos captured by themselves, photos from magazines, and so on, after which they gave their collage a title and presented their work to the group. The appreciation session followed immediately after the photography session; participants selected one of their own photographs to be projected onto a large-screen in front of the group, and positive discussions followed. In the appreciation phase that followed collage production, each participant’s collage was appreciated by all the participants and positive discussions followed.

In order to maintain positive emotions, participants performed daily homework as follows: (1) try to remember the three scenes that you enjoyed today as though it were a photographic image (reference: “three good things in life” [8,17]); (2) when you are lying in bed before sleeping, please try to recite the following words of gratitude in your mind: (1) I appreciate that I live here and now, (2) I appreciate everyone, and (3) I appreciate myself (reference: “gratitude visit” [8,17]).

Facilitators aimed to make the sessions positive and enjoyable, and used a person-centered approach [26,27], person-centered care [28], and expressive art therapy [29]. Most importantly, the facilitators tried to relay positive emotion, fun, and empathy, because emotional information can be transmitted to others via this approach [30,31,32]. The primary investigator trained the facilitators on the purpose and contents of the program, characteristics of older adults, and skills required to effectively manage the sessions.

#### 2.5.2. Photo Correspondence Education (Control Group) 

Photo Correspondence Education (PCE) acted as the control intervention. For this, a photography-related text was mailed to each participant once a week (12 times over 3 months). Participants learned the history of the photographs and photography by themselves; they were instructed to read the text for approximately 10 min per day, and were permitted to capture photographs of their choice during the PCE period.

### 2.6. Outcome Measures

The primary outcome was depressive mood. Psychological measures were selected such that the participants are least stressed. A face-to-face assessment was conducted for cognitive measurements. Demographic information (age, sex, and number of years of education) was collected using a self-report questionnaire. This information was collected twice: pre-intervention (within 3 weeks before the intervention) and post-intervention (within 1 week after the intervention), for all participants.

#### 2.6.1. Primary Outcome Measures

A Japanese version [33] of the Center for Epidemiologic Studies Depression (CES-D) Scale [34] was used to measure depressive mood. The CES-D consists of 20 items scored on a 4-point scale (0–3), with a total possible score from 0 to 60. Higher scores indicate a greater severity of depression, and a score of ≥16 indicates depression [34]. The Cronbach’s alpha coefficient for this study was 0.82 (pre-intervention [pre]) and 0.71 (post-intervention [post]).

#### 2.6.2. Secondary Outcome Measures

Psychological measurements: The Japanese version [35,36] of the Kessler 6-item Psychological Distress Scale (K-6) [37] was used to measure psychological distress. This measure has been validated [38], and assesses psychological distress in the past 30 days. It consists of 6 items rated on a 5-point scale (0–4). Total scores range from 0 to 24, with higher scores indicating higher psychological distress (Cronbach’s alpha = 0.87 [pre], 0.77 [post]).

The Japanese version [39] of the Apathy Scale [40] was used to measure apathy. This scale has been validated [39], and consists of 14 items scored on a 4-point scale (0–3). The total score ranges from 0 to 42, with higher scores indicating higher apathy. A score of ≥16 indicates apathy [39] (Cronbach’s alpha = 0.87 [pre], 0.82 [post]).

The Japanese version [41] of the Satisfaction with Life Scale (SWLS) [42] is a validated measure [41] that assesses overall life satisfaction. It consists of 5 items scored on a 7-point scale (1–7). The total scores range from 5 to 35, with higher scores indicating a greater satisfaction with life (Cronbach’s alpha = 0.91 [pre], 0.89 [post]). 

The Japanese version [43] of the Positive and Negative Affect Schedule (PANAS) [44] is a validated measure of positive (10 items) and negative (10 items) emotions [43,45]. In this study, only the positive version was used. Items are scored on a 6-point scale (1–6) based on the degree of correspondence to current feelings, with total scores ranging from 10 to 60, and higher scores indicating more positive emotion (Cronbach’s alpha = 0.91 [pre], 0.89 [post]).

Cognitive functions were assessed using the National Center for Geriatrics and Gerontology-Functional Assessment Tool (NCGG-FAT). The NCGG-FAT consists of the following domains: memory (immediate and delayed word-list memory, and immediate and delayed logical memory), attention (an electronic tablet version of the Trail Making Test-Part A [TMT-part A]), executive function (an electronic tablet version of the Trail Making Test-Part B [TMT-part B]), processing speed (an electronic tablet version of the Digit Symbol Substitution Test [DSST]), and visuospatial function (figure selection). Participants were given approximately 25 min to complete the tablet-based assessments. The NCGG-FAT has been shown to exhibit high test–retest reliability, moderate-to-high criterion-related validity, and predictive validity for dementia among community-dwelling older adults [46,47].

Other measurements and attendance and participants’ evaluation of the intervention were performed only for the PPA group.

### 2.7. Statistics

A linear mixed-effects model was used to examine the effect of the program on the primary and secondary endpoints, applying the intention-to-treat principle. The fixed and random effects were specified as follows: fixed effect: intercept, group, time, and group × time interaction; random effect: intercept. Missing values were not included in the analyses. Additionally, for the primary outcome (CES-D score), we conducted a series of sub-group analyses using the dichotomized baseline CES-D score (below/above the cut-off), age (65–74/75 years or above), and sex (male/female). Finally, we calculated the effect sizes (Cohen’s d) [48] for the adjusted mean differences between the groups. All analyses were conducted using SPSS Version 23.0 J (IBM, Armonk, NY, USA). The significance level was set at *p* < 0.05.

## 3. Results

A total of 57 people who met the inclusion criteria were enrolled. Immediately after enrollment, two participants were excluded because of serious illness before the randomization. We randomized the remaining 55 participants into two groups. Before the program started, one participant in the intervention group was lost to follow-up because of serious illness. The program was started with 54 participants (PPA: *n* = 27, PCE: *n* = 27). Immediately after the first day of the program, one participant in the PPA group was lost to follow-up because of bereavement due to death of the partner. The final analysis was performed using data from 55 participants at the pre-intervention time point and 53 at the post-intervention time point using the intention-to-treat principle (Figure 1).

### 3.1. Baseline Characteristics of Study Participants

Baseline characteristics of the PPA and PCE groups are presented in Table 1.

### 3.2. Feasibility of the PPA Program

No adverse events occurred during the intervention period. All participants were able to use the camera in the PPA group. Attendance and participants’ evaluation of the intervention is shown in Table 2.

### 3.3. Primary Outcome: CES-D Score

**Figure 2:**
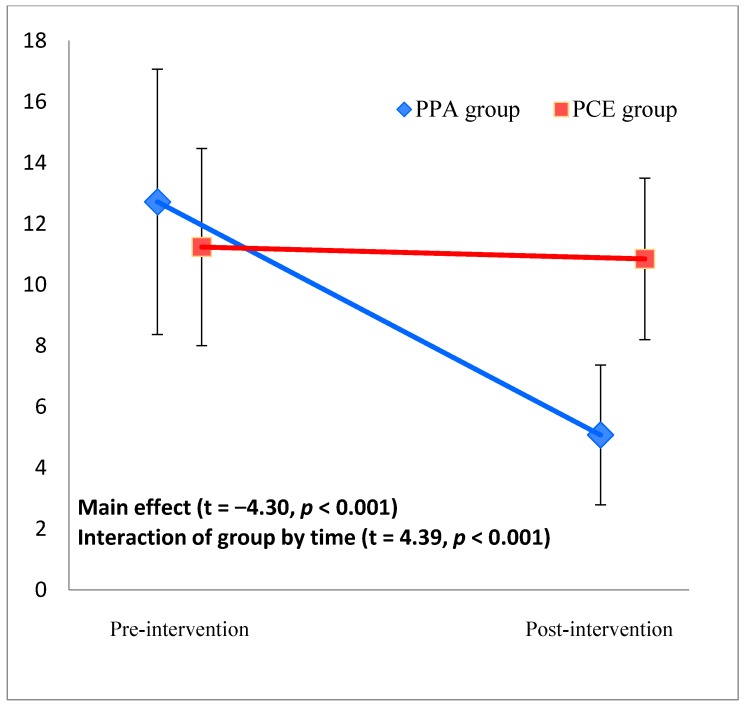
Center for Epidemiologic Studies Depression Scale (CES-D) scores in the PPA and PCE groups pre- and post-intervention. Error bars represent the standard deviation. Baseline (intervention group, *n* = 27: 12.67 ± 8.48; control group, *n* = 25: 11.24 ± 6.46) and post-intervention CES-D score (intervention group, *n* = 26: 5.08 ± 4.58; control group, *n* = 27: 10.85 ± 5.30).

A significant main effect of group (*t* = −4.30, *p* < 0.001) and interaction effect of group by time (i.e., differential effect of group by time) (*t* = 4.39, *p* < 0.001) were observed, indicating a significant improvement in the CES-D score in the PPA group compared with the PCE group (Figure 2). The group mean differences ±SD were −7.84 ± 7.21 (difference between pre-intervention and post-intervention scores; 4.88 ± 4.55–12.72 ± 8.71) for the PPA group and −0.2 ± 4.98 (11.04 ± 5.42–11.24 ± 6.46) for the PCE group, with an effect size of Cohen’s *d* = 1.23 (*p* < 0.001). The sample size calculated based on the effect size was *n* = 15. Since the sample size was sufficiently large, we performed additional sub-group analyses.

The stratified analysis by the baseline CES-D score showed similar results among the score group strata (Appendix A Table A1). In the group without depressive tendency (CES-D < 16), a significant main effect of group (*t* = −2.06, *p* < 0.001) and an interaction effect of group by time (*t* = 4.61, *p* < 0.001) were observed, indicating significantly larger improvement in the CES-D score in the PPA group than that in the PCE group. In the group with depressive tendency (CES-D ≥ 16), a significant improvement in the CES-D score was observed in the PPA group compared with the PCE group, which was shown by the interaction effect of group by time (*t* = 2.39, *p* = 0.035); however, the main effect of group (*t* = −2.06, *p* = 0.064) was marginal. Likewise, stratified analysis by age and sex showed a significant and similar improvement in the PPA group in men (main effect of group : *t* = −2.66, *p* = 0.019; interaction effect of group by time: *t* = 4.56, *p* = 0.001) and women (main effect: *t* = −3.49, *p* = 0.001; interaction effect of group by time: *t* = 2.70, *p* = 0.011), the younger group (main effect of group: *t* = −2.71, *p* = 0.011; interaction of group by time: *t* = 3.23, *p* = 0.003), and the older group (main effect of group: *t* = −3.40, *p* = 0.003; interaction effect of group by time: *t* = 3.55, *p* = 0.002).

### 3.4. Secondary Outcomes

There was a significant interaction effect of group by time on the PANAS score (*t* = −2.33, *p* = 0.024), but the main effect of group was only marginal (*t* = 1.78, *p* = 0.080). No other significant between-group differences were found in the other variables (Table 3).

## 4. Discussion

We developed the PPA program based on a combination of the multiple dimensions of PPT that improve depressive symptoms by enhancing positive emotions, engagement, and meaning [8]. To the best of our knowledge, the present study is the first to examine the effect of a positive psychological intervention program on depressive mood improvement in older adults using a pilot RCT design. The results show that the primary outcome (depressive mood) significantly improved in the PPA group compared with the PCE group. Additionally, positive emotions, assessed based on the PANAS score (one of the secondary outcomes), also showed a significant improvement. These findings are consistent with our hypothesis.

Furthermore, our study confirmed the feasibility of the PPA program. There were no adverse events during the intervention period. In addition, all participants in the intervention group were able to use the camera without any problem. Moreover, over 80% of the participants showed perfect attendance and were fully satisfied with the PPA program.

The results show a relatively large effect size (Cohen’s *d* = 1.23) of the primary outcome. This effect size was equivalent to that of the depression scale score found previously (*d* = 1.12) following a PPT used in a pilot RCT [8]. Another PPT pilot study also reported a large effect size of mood improvement: depression scale, *d* = 0.90 and CES-D, *d* = 0.93 [49]. The high participation rate and enjoyment for the program observed in this study may have contributed to the relatively large effect size. In addition, a larger effect on mood improvement may be observed with the PPA program due to the multiple components of photography, collage production, appreciation, group discussion, facilitation, and homework (based on the three positive psychology elements) compared with the PCE program. Moreover, the stratified analysis by depressive tendency, age, or sex also showed similar findings. These results suggest that the PPA program could, therefore, have the potential to alleviate depressive symptoms, regardless of the depression tendency, age group, or sex (Table A1).

As secondary outcomes, the present study also examined the changes before and after the administration of psychological assessments and cognitive tests. Results indicated an increased positive emotion (PANAS) similar to that observed in previous studies on other positive psychological interventions [50,51]. However, other psychological measures, such as psychological distress (K-6), apathy, and SWLS, did not indicate significant differences, contrary to our hypothesis. This implies that the PPA program responds to short-term affective fluctuations in mood [44] rather than life satisfaction by a long-term perspective [42].

We also examined whether PPA made additional contributions to improved cognitive function outcomes based on our hypothesis. Unfortunately, we found no significant changes in cognitive functioning. Therefore, this study does not support the hypothesis that cognitive function improves with mood improvement. This could be because a three-month program may be insufficient for any improvement in cognitive functions. Additionally, this study targeted healthy older adults; cognitive function tests can often be affected by biases due to a floor or ceiling effect, depending on the baseline score of the healthy participants [52,53]. Overall, however, these results suggest that the developed PPA program could be a promising intervention for alleviating depressive mood in community-dwelling healthy older adults.

There are several limitations to this study. First, since the study participants were limited to a small number of healthy Japanese elderly individuals who lived in two municipalities, the generalizability of this study is limited. However, the mean CES-D score of the study participants at baseline was similar to those reported in a large-scale cohort study that targeted community-dwelling older adults [54]. Second, as we used a combination approach in anticipation of a large effect, the respective effect of for positive emotion, engagement, and meaning are unclear. Moreover, we could not assess the effect of photography per se in this study. Future studies should examine the specific effects of each of these dimensions. Third, this study did not include a follow-up examination after intervention. Therefore, it could not show whether there was a sustained effect of PPA.

## 5. Conclusions

Our results show that the PPA program could decrease depressive mood with a relatively large effect size. Based on these results, we will proceed to the next investigation using an RCT design. The findings of the present study contribute to the development of PPI, as well as other psychological interventions, by demonstrating the effect using a relatively robust design for the alleviation of depressive mood in older adults. This approach could be a possible option among non-pharmacological programs for the prevention of depressive symptoms in older adults.

## Figures and Tables

**Table 1:** Pre-intervention characteristics of the study participants (*n* = 55).

Measurement ^a^		PPA ^b^ Group	*n*	PCF ^b^ Group	*n*	*p*-Value
Sociodemographic characteristics						
Age (in years)	Mean ± SD	74.29 ± 5.87	28	73.04 ± 5.01	27	0.847
Sex (female)	*n* (%)	20 (71.4%)	28	19 (70.4%)	27	1.00
Education (in years)	Mean ± SD	12.00 ± 2.71	28	12.22 ± 2.41	27	0.749
Psychological measurements						
CES-D	Mean ± SD	12.67 ± 8.48	27	11.24 ± 6.46	25	0.501
K-6	Mean ± SD	3.00 ± 3.19	28	3.35 ± 3.44	26	0.703
Apathy Scale	Mean ± SD	11.13 ± 5.40	23	11.42 ± 5.61	24	0.860
SWLS	Mean ± SD	24.29 ± 5.87	28	23.63 ± 6.25	27	0.690
PANAS	Mean ± SD	34.40 ± 7.76	25	34.20 ± 6.46	25	0.922
Cognitive measurements						
DSC_90	Mean ± SD	46.57 ± 8.89	28	43.89 ± 10.08	27	0.299
DSC_120	Mean ± SD	63.60 ± 12.08	28	59.63 ± 13.64	27	0.257
FS	Mean ± SD	5.89 ± 1.23	28	6.04 ± 1.26	27	0.688
ADAS1	Mean ± SD	7.74 ± 0.96	28	7.98 ± 1.15	27	0.411
ADAS2	Mean ± SD	4.00 ± 2.11	28	4.11 ± 2.69	27	0.865
ADAS3	Mean ± SD	7.50 ± 1.20	28	7.52 ± 1.89	27	0.965
TMT_A	Mean ± SD	25.25 ± 19.73	28	24.89 ± 13.24	27	0.937
TMT_B	Mean ± SD	35.36 ± 12.86	28	44.88 ± 30.41	27	0.142
SM1	Mean ± SD	6.64 ± 1.91	28	7.22 ± 2.01	27	0.277
SM2	Mean ± SD	7.18 ± 1.79	28	7.52 ± 1.70	27	0.473

Notes: ^a^: CES-D: Center for Epidemiologic Studies Depression Scale; K-6: Kessler 6-item Psychological Distress Scale; Apathy: Apathy Scale, SWLS: Satisfaction with Life Scale; PANAS: Positive and Negative Affect Schedule; DSC_90 and DSC_120: Digit Symbol-Coding subtest of the Wechsler Adult Intelligence Scale III; FS: Figure selection; ADAS1, ADAS2, and ADAS3: Word recognition subtest of The Alzheimer’s Disease Assessment Scale—Cognitive Subscale; TMT_A and B: The tablet version of the Trail Making Test—parts A and B, respectively; SM1: Story memory-I, immediate recognition; SM2: Story memory-II, delayed recognition; n: Number, F: Female. ^b^: PPA and PCE indicate Positive Photo Appreciation and Photo Correspondence Education, respectively.

**Table 2:** Attendance and participants’ evaluation of the intervention.

**Attendance**	
Perfect attendance	82.1%
Absent once	7.1%
7 times absent	3.6%
Lost to follow up: owing to a serious illness	3.6%
Lost to follow up: owing to the bereavement due to death of partner	3.6%
**Evaluation of Participants**	
Very enjoyable	82.1%
Enjoyable	7.1%
Normal	3.6%
Not enjoyable	0%
Not very enjoyable	0%
Lost to follow up: owing to a serious illness	3.6%
Lost to follow up: owing to the bereavement due to death of partner	3.6%

Note: Analysis was performed using the intention-to-treat principle, and included the participants who were lost to follow-up.

**Table 3:** Secondary outcomes pre- and post-intervention in the positive photo appreciation (PPA) and photo correspondence education (PCE) groups.

Measurement ^a^	Group	Pre-Intervention		Post-Intervention		Main Effect (Group)		Interaction Effect (Group by Time)	
		Mean ± SD	*n*	Mean ± SD	*n*	*t*	*p*	*t*	*p*
Psychological measurements									
K-6	PPA	3.00 ± 3.19	28	2.21 ± 2.77	24	−1.50	0.141	1.10	0.276
	PCE	3.35 ± 3.44	26	3.22 ± 2.89	27				
Apathy	PPA	11.13 ± 5.40	23	9.32 ± 4.71	25	−1.64	0.107	0.92	0.362
	PCE	11.42 ± 5.61	24	11.32 ± 4.81	25				
SWLS	PPA	24.29 ± 5.87	28	25.31 ± 6.66	26	0.49	0.624	−0.11	0.917
	PCE	23.63 ± 6.25	27	24.56 ± 5.15	27				
PANAS	PPA	34.40 ± 7.76	25	40.92 ± 7.33	24	1.79	0.080	−2.33	0.024
	PCE	38.12 ± 6.85	25	37.22 ± 7.37	27				
Cognitive measurements									
DSC_90	PPA	46.57 ± 8.89	28	48.65 ± 10.62	26	1.26	0.212	−0.65	0.520
	PCE	43.89 ± 10.08	27	45.29 ± 10.33	27				
DSC_120	PPA	63.60 ± 12.08	28	65.73 ± 13.77	26	1.25	0.217	−0.38	0.703
	PCE	59.63 ± 13.64	27	61.22 ± 13.91	27				
FS	PPA	5.89 ± 1.23	28	5.85 ± 0.97	26	0.43	0.668	−0.64	0.523
	PCE	6.04 ± 1.26	27	5.70 ± 1.35	27				
ADAS1	PPA	7.74 ± 0.96	28	7.87 ± 10.62	26	-0.67	0.509	−0.11	0.914
	PCE	7.98 ± 1.15	27	8.05 ± 1.11	27				
ADAS2	PPA	4.00 ± 2.11	28	5.31 ± 1.69	26	0.82	0.411	−1.08	0.287
	PCE	4.11 ± 2.69	27	4.89 ± 2.17	27				
ADAS3	PPA	7.50 ± 1.20	28	7.31 ± 1.67	26	−0.53	0.602	0.51	0.611
	PCE	7.52 ± 1.89	27	7.56 ± 2.06	27				
TMT_A	PPA	25.25 ± 19.73	26	19.69 ± 4.40	26	− ^b^	0.942 ^b^	− ^b^	0.727 ^b^
	PCE	24.89 ± 13.24	27	20.56 ± 5.24	27				
TMT_B	PPA	35.36 ± 12.86	28	36.15 ± 14.78	26	−1.31	0.194	0.06	0.956
	PCE	44.88 ± 30.41	27	45.96 ± 35.96	27				
SM1	PPA	6.64 ± 1.91	28	8.35 ± 0.98	26	0.39	0.695	−1.84	0.072
	PCE	7.22 ± 2.01	27	8.19 ± 1.78	27				
SM2	PPA	7.18 ± 1.79	28	8.30 ± 1.35	26	0.29	0.776	−1.38	0.173
	PCE	7.51 ± 1.70	27	8.15 ± 1.77	27				

Notes: ^a^: CES-D: Center for Epidemiologic Studies Depression Scale; K-6: Kessler 6-item Psychological Distress scale; Apathy: Apathy Scale; SWLS: Satisfaction with Life Scale; PANAS: Positive and Negative Affect Schedule; DSC_90 and DSC_120: Digit Symbol-Coding subtest of the Wechsler Adult Intelligence Scale III; FS: Figure selection; ADAS1, ADAS2, and ADAS3: Word recognition subtest of The Alzheimer’s Disease Assessment Scale—Cognitive Subscale; TMT_A and B: The tablet version of the Trail Making Test—parts A and B, respectively; SM1: Story memory-I, immediate recognition; SM2: Story memory-II, delayed recognition; *n*: Number, F: Female. ^b^: TMT-A was analyzed using a repeated general linear model instead of a linear mixed model due to software limitations.

## Appendix A

**Table A1:** Stratified analysis of CES-D ^a^ score change by sex, age group, and baseline CES-D ^a^ score.

Measurement	Group	Pre-Intervention		Post-Intervention		Measurement (Group)		Interaction Effect (Group by Time)	
		Mean ± SD	*n*	Mean ± SD	*n*	t	*p*	t	*p*
Male	PPA	11.43 ± 3.40	7	3.13 ± 3.23	8	−2.66	0.019	4.56	0.001
	PCE	7.13 ± 4.91	8	9.00 ± 5.35	8				
Female	PPA	13.10 ± 9.70	20	5.94 ± 4.89	17	−3.49	0.001	2.70	0.011
	PCE	13.17 ± 2.01	18	11.63 ± 5.22	19				
Younger	PPA	14.07 ± 9.72	15	5.87 ± 4.93	15	−2.71	0.011	3.23	0.003
	PCE	10.06 ± 6.22	16	10.29 ± 4.22	17				
Older	PPA	10.92 ± 6.63	12	4.00 ± 3.92	11	−3.40	0.003	3.55	0.002
	PCE	13.33 ± 6.71	9	11.80 ± 6.91	10				
Non Depression ^b^	PPA	8.95 ± 4.21	20	3.67 ± 2.61	19	−4.78	<0.001	4.61	<0.001
	PCE	8.28 ± 4.85	18	9.22 ± 4.70	18				
Depression ^b^	PPA	23.29 ± 8.85	7	9.67 ± 6.25	6	−2.06	0.064	2.39	0.035
	PCE	18.86 ± 2.54	7	15.71 ± 4.42	7				

PPA: Positive Photo Appreciation, PCE: Photo Correspondence Education. Note: ^a^: CES-D, Center for Epidemiologic Studies Depression Scale. ^b^: Respondents in the non-depression group had a baseline CES-D score of 16 points or less. Those in the depression group had a baseline CES-D score above 16 points.
